# Nine species from Madagascar are moved from *Vernonia* to *Distephanus* (Compositae, Vernonieae)

**DOI:** 10.3897/phytokeys.77.11727

**Published:** 2017-03-17

**Authors:** Vicki A. Funk, Harold Robinson

**Affiliations:** 1 Department of Botany, MRC 166, National Museum of Natural History, Smithsonian Institution, Washington, DC. 20023-7012

**Keywords:** Asteraceae, Asterids, flowering plants, Madagascar, Vernonia

## Abstract

The genus *Distephanus* is native to Madagascar, the Mauritius, central and southern Africa, Yemen (Socotra Island), and China. The majority of the diversity is found in Madagascar. Here we provide new combinations for nine species of *Vernonia* that belong in *Distephanus*, all from Madagascar. All of the species were formerly placed in the large genus *Vernonia*, now greatly reduced.

## Introduction

As we continue to emerge from the “Dark Ages of Lumping” it should come as no surprise to any student of the Compositae that morphological and molecular data are being used (separately and together) to address generic limits. As a result we are seeing the breakup of many large non-monophyletic genera. In one such case, the genus *Vernonia* Schreb., of the tribe Vernonieae, has shrunk to about 20 species from North America and the remaining 1000 or so species from that genus are in the process of being assigned to other genera. Many of these “new” genera were previously described and subsequently sunk into *Vernonia*, but others needed new names and descriptions. An overview of the tribe was presented by Keeley and Robinson (2009) and major overhauls have taken place for the Americas (Robinson 1999), China (Robinson and Skvarla 2010), Thailand (Bunwong et al. 2014) and Southern Africa (Swelankomo and Manning 2014; Robinson et al. 2016). Still awaiting work are the Vernonieae of Madagascar, tropical Africa, India, and SE Asia.

As part of a larger more comprehensive work on the Vernonieae of Madagascar this effort concerns the establishment of proper limits for the genus *Distephanus* Cass. Described by Cassini based on a type removed from *Conyza* (Astereae), *Distephanus* has long been recognized as distinctive (trees, shrubs or woody vines; yellow or orange flowers; tri-nervate leaf venation) even when it was considered part of *Vernonia* (herbs or subshrubs; purple, pink or white flowers; pinnate leaf venation). In addition to the type species three other species were described in *Distephanus* and therefore do not need to be transferred and a number of combinations have already been made: 24 by Robinson and Kahn (1986), one by Robinson (2009), two by Robinson (2012), one by Robinson and Funk (in Funk et al. 2012) and one by Boon and Glen (2013) for a total of 33 species currently in *Distephanus*. However, because of a lack of available herbarium material some of the Madagascar species names were left as *Vernonia*. After examining herbarium material from the Muséum National d’Histoire Naturelle (P), nine additional combinations can now be made. The following combinations are needed at this time because of upcoming entries in GenBank and determinations on specimens from a recent field trip in September-October of 2016. Descriptions and synonomy can be found in Humbert (1960: Volume 1, 121–171). With these additions, the number of species in *Distephanus* now stands at 42 with possible new species to be described based on material collected during the aforementioned field trip.

## *Vernonia* species from Madagascar transferred into *Distephanus*

### Distephanus bakeri

Taxon classificationPlantaeAsteralesAsteraceae

(Vatke) V.A.Funk & H.Robinson comb. nov.

urn:lsid:ipni.org:names:77161439-1

Vernonia bakeri Vatke. Bremen Abh. Natuewiss. Vereins Bremen 9: 119. 1885.

### Distephanus capuronii

Taxon classificationPlantaeAsteralesAsteraceae

(Humbert) V.A.Funk & H.Robinson comb. nov.

urn:lsid:ipni.org:names:77161440-1

Vernonia capuronii Humbert, Mem. Inst. Sci. Madagascar, Sér. B., Bíol. Veg. 6: 152. 1955.

### Distephanus grevei

Taxon classificationPlantaeAsteralesAsteraceae

(Drake) V.A.Funk & H.Robinson comb. nov.

urn:lsid:ipni.org:names:77161442-1

Vernonia grevei Drake. Bull. Soc. Bot. France 46: 240. 1900 [dt. 1899; publ. early1900]

### Distephanus ibityensis

Taxon classificationPlantaeAsteralesAsteraceae

(Humbert) V.A.Funk & H.Robinson comb. nov.

urn:lsid:ipni.org:names:77161443-1

Vernonia ibityensis Humbert, Notul. Syst. (Paris) 13(4): 313. 1949 [dt. Apr 1948; publ. early 1949]

### Distephanus poissonii

Taxon classificationPlantaeAsteralesAsteraceae

(Humbert) V.A.Funk & H.Robinson comb. nov.

urn:lsid:ipni.org:names:77161444-1

Vernonia poissonii Humbert, Notul. Syst. (Paris) 8(1): 6. 1939.

### Distephanus polytricholepis

Taxon classificationPlantaeAsteralesAsteraceae

(Baker) V.A.Funk & H.Robinson comb. nov.

urn:lsid:ipni.org:names:77161445-1

Vernonia polytricholepis Baker, J. Linn. Soc., Bot. 21: 415. 1885

### Distephanus quartziticola

Taxon classificationPlantaeAsteralesAsteraceae

(Humbert) V.A.Funk & H.Robinson comb. nov.

urn:lsid:ipni.org:names:77161446-1

Vernonia quartziticola Humbert, Notul. Syst. (Paris) 13(4): 305. 1949 [dt. Apr 1948; publ. early 1949]

### Distephanus rhodopappus

Taxon classificationPlantaeAsteralesAsteraceae

(Baker) V.A.Funk & H.Robinson comb. nov.

urn:lsid:ipni.org:names:77161447-1

Vernonia rhodopappa Baker. J. Linn. Soc., Bot. 22: 487. 1887

### Distephanus spiciformis

Taxon classificationPlantaeAsteralesAsteraceae

(Klatt) V.A.Funk & H.Robinson comb. nov.

urn:lsid:ipni.org:names:77161448-1

Vernonia spiciformis Klatt. Ann. Nat. Hofmus. Wien 7: 296. 1892

## Supplementary Material

XML Treatment for Distephanus bakeri

XML Treatment for Distephanus capuronii

XML Treatment for Distephanus grevei

XML Treatment for Distephanus ibityensis

XML Treatment for Distephanus poissonii

XML Treatment for Distephanus polytricholepis

XML Treatment for Distephanus quartziticola

XML Treatment for Distephanus rhodopappus

XML Treatment for Distephanus spiciformis

## References

[B1] Boon R Glen HF (2013) *Distephanus* (Asteraceae: Vernonieae): a new combination and a new record for southern Africa. Bothalia 43(1): 94–96. https://archive.org/stream/bothaliavolume4343unse/bothaliavolume4343unse_djvu.txt

[B2] Bunwong S Chantaranothai P Keeley SC (2014) Revision and key to the Vernonieae of Thailand. Phytokeys 37: 25–101. https://doi.org/10.3897/phytokeys.37.6499 10.3897/phytokeys.37.6499 PMC4023333 24843297

[B3] Funk VA Kelloff C Chan R (2012) Phylogeny and Biogeography of the Tribe Liabeae (Compositae; subfamily Cichoroideae). Taxon 61: 437–455. https://www.researchgate.net/publication/247150678_Phylogeny_and_Biogeography_of_the_Tribe_Liabeae_Compositae_subfamily_Cichoroideae

[B4] Humbert H (1960) Flore de Madagascar et des Comores. 189^e^ Famille – Composees, vol. 1. Typographie Firmin-Didot, Paris. http://biodiversitylibrary.org/page/8108583

[B5] Keeley SC Robinson H (2009) Vernonieae. In: Funk VA Susanna A Stuessy TF Bayer RJ (Eds) Systematics, Evolution, and Biogeography of Compositae. International Association for Plant Taxonomy, Vienna, 439–469.

[B6] Robinson H Kahn B (1986) Trinervate leaves, yellow flowers, tailed anthers, and pollen variation in *Distephanus* Cassini (Vernonieae: Asteraceae). Proceeding of the Biological Society of Washington 99: 493–501. https://doi.org/10.1098/rspb.1986.0036

[B7] Robinson H (1999) Generic and Subtribal classification of American Vernonieae. Smithsonian Contributions to Botany 89: 1–116. https://doi.org/10.5962/bhl.title.103697

[B8] Robinson H (2009) The generic disposition of the African *Vernonia biafrae* Oliv. & Hiern. (Vernonieae: Asteraceae). Phytologia 91(3): 534–536. http://biodiversitylibrary.org/page/28528804

[B9] Robinson H Skvarla JJ (2010) Genera of the Vernonieae (Asteraceae) of China with a study of their pollen. Taiwania 55(3): 254–272. https://doi.org/10.6165/tai.2010.55(3).254

[B10] Robinson H Skvarla JJ Funk VA (2016) Vernonieae (Asteraceae) of Southern Africa: A generic disposition of the species and a study of their pollen. Phytokeys 60: 49–126. https://doi.org/10.3897/phytokeys.60.6734 10.3897/phytokeys.60.6734 PMC4816990 27081344

[B11] Robinson H (2012) Two new combinations in the genus *Distephanus* Cass. (Asteraceae, Vernonieae). PhytoKeys 17: 25–26. https://doi.org/10.3897/phytokeys.17.4013 10.3897/phytokeys.17.4013 PMC3519351 23233815

[B12] Swelankomo N Manning JC (2014) The genus *Distephanus* (Asteraceae: Vernonieae) in southern Africa. South African Journal of Botany 94: 238–248. https://doi.org/10.1016/j.sajb.2014.07.007

